# La nécessité des approches interdisciplinaires et collaboratives pour évaluer l’impact de la COVID-19 sur les personnes âgées et le vieillissement: déclaration conjointe de l’ACG / CAG et de la RCV / CJA

**DOI:** 10.1017/S071498082000032X

**Published:** 2020-08-12

**Authors:** Brad A. Meisner, Veronique Boscart, Pierrette Gaudreau, Paul Stolee, Patricia Ebert, Michelle Heyer, Laura Kadowaki, Christine Kelly, Mélanie Levasseur, Ariane S. Massie, Verena Menec, Laura Middleton, Linda Sheiban Taucar, Wendy Loken Thornton, Catherine Tong, Deborah K. van den Hoonaard, Kimberley Wilson

**Affiliations:** 1 Conseil d’administration, Association canadienne de gérontologie/Canadian Association on Gerontology; 2 Comité de rédaction, Revue canadienne du vieillissement/Canadian Journal on Aging; 3 School of Kinesiology and Health Science, York University; 4 School of Health and Life Sciences/Schlegel Centre for Advancing Seniors Care, Conestoga College; 5 Département de médecine, Université de Montréal; 6 School of Public Health and Health Systems, University of Waterloo; 7 Seniors Health Services, Alberta Health Services/Department of Psychology; 8 Connexion étudiante, Association canadienne de gérontologie/Canadian Association on Gerontology; 9 Department of Gerontology, Simon Fraser University; 10 Department of Community Health Sciences, University of Manitoba; 11 École de réadaptation, Université de Sherbrooke; 12 Department of Kinesiology, University of Waterloo; 13 Department of Psychology, Simon Fraser University; 14 Department of Gerontology, St. Thomas University; 15 Department of Family Relations and Applied Nutrition, University of Guelph

**Keywords:** vieillissement, coronavirus, relations interprofessionnelles, communication scientifique, recherche interdisciplinaire, collaboration intersectorielle, aging, coronavirus, interprofessional relations, scholarly communication, interdisciplinary research, intersectoral collaboration

## Abstract

La pandémie de la COVID-19 et l’état d’urgence publique qui en a découlé ont eu des répercussions significatives sur les personnes âgées au Canada et à travers le monde. Il est impératif que le domaine de la gérontologie réponde efficacement à cette situation. Dans la présente déclaration, les membres du conseil d’administration de l’Association canadienne de gérontologie/Canadian Association on Gerontology (ACG/CAG) et ceux du comité de rédaction de *La Revue canadienne du vieillissement*/*Canadian Journal on Aging* (*RCV*/*CJA*) reconnaissent la contribution des membres de l’ACG/CAG et des lecteurs de la *RCV*/*CJA.* Les auteurs exposent les voies complexes par lesquelles la COVID-19 affecte les personnes âgées, allant du niveau individuel au niveau populationnel. Ils préconisent une approche impliquant des équipes collaboratives pluridisciplinaires, regroupant divers champs de compétences, et différentes perspectives et méthodes d’évaluation de l’impact de la COVID-19.

## Abstract (en)

The COVID-19 pandemic and subsequent state of public emergency have significantly affected older adults in Canada and worldwide. It is imperative that the gerontological response be efficient and effective. In this statement, the board members of the Canadian Association on Gerontology/L’Association canadienne de gérontologie (CAG/ACG) and the *Canadian Journal on Aging*/*La revue canadienne du vieillissement* (*CJA*/*RCV*) acknowledge the contributions of CAG/ACG members and *CJA*/*RCV* readers. We also profile the complex ways that COVID-19 is affecting older adults, from individual to population levels, and advocate for the adoption of multidisciplinary collaborative teams to bring together different perspectives, areas of expertise, and methods of evaluation in the COVID-19 response.

## Introduction

La pandémie de COVID-19 a entraîné de multiples changements sans précédent à travers le Canada. Les restrictions gouvernementales en matière de mobilité, la fermeture des lieux de travail non essentiels, les recommandations concernant la distanciation physique et l’isolement, la virtualisation du travail et de l’éducation, ainsi que la demande accrue de services de soins de santé essentiels ont considérablement transformé la vie quotidienne des Canadiens et des citoyens à l’étranger. Bien que la COVID-19 nous affecte tous, les personnes âgées sont les plus durement touchées par cette pandémie qui a mis en lumière certains défis connus, mais aussi d’autres défis non reconnus auxquels cette population est confrontée. Il est urgent d’élaborer des réponses efficaces aux impacts de la COVID-19 sur les personnes âgées au Canada et à travers le monde. En tant qu’organisations nationales scientifiques, éducatives et de diffusion de premier plan sur les questions relatives aux personnes âgées et au vieillissement au Canada, l’Association canadienne de gérontologie/Canadian Association on Gerontology (ACG/CAG) et *La revue canadienne du vieillissement/Canadian Journal on Aging* (*RCV/CJA*) ont produit cette déclaration en se basant sur deux objectifs clés.

En premier lieu, nous tenons à reconnaître l’implication de nos membres de l’ACG/CAG et de nos lecteurs du *RCV/CJA.* *Merci.* En tant que chercheurs, professionnels, organisations, étudiants, soignants œuvrant activement dans divers milieux, ou comme personnes âgées au Canada, membres de l’ACG/CAG et lecteurs du *RCV/CJA*, plusieurs d’entre vous ont travaillé directement ou indirectement avec les personnes âgées pendant cette pandémie. Ces activités professionnelles, qui aujourd’hui revêtent une importance accrue, se déroulent dans des conditions difficiles au sein et à travers de multiples secteurs. Nous tenons à exprimer notre gratitude pour vos contributions aux institutions universitaires et à l’enseignement supérieur, à la pratique et à l’administration des soins de santé, aux soins et au développement communautaires, ainsi qu’à la politique publique et à la gouvernance durant la pandémie de COVID-19. Pendant que vous poursuivrez vos activités, nous nous engageons à vous communiquer des informations et des résultats probants, ainsi que des occasions d’engagement, de discussion et de collaboration, soit par courrier électronique, par les médias sociaux, des sites web, des webinaires et des publications à venir sur la COVID-19.

En second lieu, nous voulons souligner les impacts complexes et multiples de la COVID-19 sur les personnes âgées, tant sur le plan individuel que sur le plan populationnel, en abordant le SRAS-CoV-2 non seulement comme un agent infectieux, mais aussi dans le contexte sociétal et historique marquant la pandémie. Aucune discipline ne pourra, à elle seule, cerner pourquoi, comment et dans quelle mesure les personnes âgées sont et seront affectées par la COVID-19. C’est pourquoi nous encourageons fortement l’adoption d’approches interdisciplinaires dans la réponse à la COVID-19, en raison de la valeur ajoutée que des liens établis entre les différentes disciplines et parmi celles-ci offrent. Afin d’illustrer la nécessité de perspectives interdisciplinaires, nous identifierons et mettrons en évidence, dans les sections suivantes, quelques exemples de questions importantes et d’opportunités à saisir en lien avec la COVID-19 en gérontologie. Ces points visent à stimuler la réflexion, ainsi qu’à susciter des questions et des programmes de recherche en lien avec la COVID-19, les personnes âgées et le vieillissement.

## Défis et opportunités pour le soutien de la santé psychologique et du bien-être

### Patricia Ebert et Wendy Loken Thornton

Pour un grand nombre de personnes âgées, la COVID-19 entraîne des défis psychologiques particuliers, incluant notamment l’isolement social accru et l’apparition ou l’exacerbation de problèmes de santé mentale, tels que le stress, l’abus d’alcool ou de drogues, l’anxiété et la dépression. Bien que de récents sondages révèlent que les personnes âgées sont moins susceptibles que les jeunes adultes de rapporter des inquiétudes ou du stress associés à la COVID-19 (Angus Reed Institute, 2020 ; Panchal et al., 2020), ce constat doit être considéré dans le contexte de l’état de santé mentale et sociale préexistant des Canadiens plus âgés. Par exemple, environ 15 % des personnes âgées au Canada n’ont pas d’amis proches (Turcotte, 2015), 25 % des personnes âgées de 85 ans et plus souffrent de solitude (Gilmour, 2012), jusqu’à 15 % des personnes âgées ont des symptômes importants de dépression (Canadian Psychological Association, 2015) et le segment des 55 ans et plus présente le deuxième taux de prévalence le plus élevé pour les troubles de comportement et les troubles anxieux (McRae et al., 2016). Les troubles mentaux réactifs ou préexistants peuvent être exacerbés chez les personnes âgées en raison de l’intense crainte de contracter la COVID-19 (c.-à-d. la coronaphobie) (Asmundson & Taylor, 2020), et celles qui présentent des troubles physiques préexistants (p. ex. MPOC, obésité) peuvent éprouver une détresse existentielle et des inquiétudes plus intenses concernant le décès et la mort.

L’exacerbation du stress des personnes âgées, des soignants et des prestataires de soins constitue aussi une problématique importante, étant donné que de nombreuses ressources de soutien à domicile, du milieu communautaires ou appuyant les personnes atteintes de démence (p. ex. programmes de jour) sont réduites, annulées ou modifiées, et fonctionnent maintenant en ligne. Le recours accru aux services virtuels ou en ligne peut toutefois constituer un obstacle. Malgré les données indiquant que les personnes âgées qui utilisent la technologie du vidéoclavardage ont un taux de dépression plus faible (Teo, Markwardt, & Hinton, 2019), la population plus âgée est aussi, en proportion, moins connectée à l’internet (Statistique Canada, 2019), et dispose de moins de connaissances, de compétences et de la confiance nécessaires pour utiliser efficacement les technologies émergentes, particulièrement en cas de déclin cognitif (Wild et al., 2012). Cette limite d’efficacité relative en matière technologique peut également contribuer à accroître la vulnérabilité aux arnaques et aux fraudes financières, qui se sont accrues depuis les débuts de la pandémie, et qui ont accentué la victimisation des personnes seules et socialement isolées (Canadian Anti-Fraud Centre, 2020). Par conséquent, des solutions pragmatiques sont nécessaires afin d’améliorer la santé mentale et d’atténuer l’isolement et la victimisation des personnes âgées.

Par ailleurs, des préoccupations ont été rapportées concernant un éventuel déclin cognitif résultant d’interventions visant à sauver des vies chez des patients avec COVID-19. De nombreux patients plus âgés requièrent une assistance respiratoire prolongée, et ceci peut entraîner des conséquences sur la santé physique et cognitive par le syndrome de post-traitement intensif (Stam, Stucki, & Bickenbach, 2020). Ces conséquences comprennent notamment la dépression, ainsi que les troubles de la mémoire, de l’attention et des atteintes d’autres fonctions neuropsychologiques (Pandharidpande et al., 2013 ; Stam et al., 2020). Ces enjeux cognitifs nécessiteront des initiatives en matière de formation pour le personnel œuvrant dans les soins psychologiques et en réadaptation avec des patients âgés ayant survécu à la COVID-19. Des stratégies fondées sur des données probantes devront être développées par les psychologues afin d’aider les patients âgés à surmonter des problèmes de santé mentale émergents et persistants, ce qui inclut aussi les troubles associés à des difficultés cognitives (Gallagher, McLeod, & McMillan, 2019). Il est nécessaire de mettre au point des échelles pour évaluer l’impact cognitif et neuropsychiatrique des mesures de contrôle visant à prévenir les infections de COVID-19 (p. ex. distanciation physique). Il sera désormais important d’intégrer le dépistage, l’évaluation et la gestion des troubles cognitifs et neuropsychologiques dans les soins psychologiques dispensés aux personnes âgées.

## Défis et opportunités pour le soutien de comportements liés à la santé et au bien-être

### Laura Middleton

Le changement comportemental rapide qui a été requis en matière de santé publique pour répondre à la COVID-19 engendrera probablement des conséquences négatives sur la santé, à court et à long terme. Les Canadiens plus âgés font maintenant face à des restrictions de leur mobilité communautaire, à une perte de soutien social et à un accès réduit aux produits et services. Plusieurs programmes communautaires qui soutenaient le bien-être social, mental et physique ont été supprimés ou fortement restreints. Il est probable que les comportements quotidiens sédentaires, associés à des risques accrus de mortalité d’origine cardiovasculaire ou autre, augmenteront (Biswas et al., 2015). Les défis de l’exercice à la maison sont encore plus importants pour de nombreuses personnes, par manque de soutien social, d’encouragement et de contrôle, des éléments facilitateurs clés pour l’activité physique (Schutzer & Graves, 2004). De plus, les recommandations visant la réduction ou l’évitement du magasinage pourraient affecter l’accès à une alimentation saine. L’isolement social et la solitude associés à la distanciation physique sont de forts prédicteurs de la morbidité et de la mortalité à un âge avancé, bien que les trajectoires en ce sens soient complexes (Freedman & Nicolle, 2020). En raison de la COVID-19, les facteurs de risque modifiables et liés au mode de vie pour les maladies chroniques, les déficiences fonctionnelles et la mortalité risquent d’être accentués.

D’une perspective plus optimiste, de plus en plus de personnes, d’organisations et d’entreprises proposent des solutions novatrices pour soutenir la santé et le bien-être des personnes âgées vivant dans la communauté. Plusieurs cas de voisins, d’étudiants et de jeunes qui soutiennent actuellement des personnes âgées se sont fait connaître. Par exemple, « Chatting to Wellness » est une organisation à but non lucratif gérée par des étudiants qui offre des liens sociaux aux personnes âgées. Ses activités se poursuivent par téléphone depuis la pandémie de COVID-19 (Jackson, 2020). Les organisations communautaires et les entreprises telles que le YMCA, la Société Alzheimer et les studios de yoga locaux se tournent vers la programmation en ligne, les bulletins et les ressources envoyés par courriel, ainsi que vers les programmes et les groupes de soutien sur le web. Bien que les personnes âgées soient généralement la population la moins encline à adopter de nouvelles technologies telles que les ordinateurs et les téléphones intelligents (Vogels, 2019), la pandémie de COVID-19 pourrait les inciter à considérer ces équipements (Finn, 2020), et ce, même chez les individus souffrant de troubles cognitifs, en particulier lorsqu’ils sont soutenus par des membres de leur famille (Wild et al., 2012). Toutefois, le manque d’équipement et d’accès à l’internet, spécialement dans les zones rurales et isolées, sont des barrières qui posent encore de grands défis. Par conséquent, les bénéfices de ces outils technologiques risquent d’être uniquement accessibles aux deux tiers de la population plus âgée utilisant déjà l’internet (Anderson & Perrin, 2017) et aux deux cinquièmes disposant de téléphones intelligents (Vogels, 2019). Il sera important d’examiner comment les leçons apprises et les ressources développées en réponse aux effets négatifs de la COVID-19 sur les comportements liés à la santé pourraient servir à la mise en place et à la pérennité des programmes et des services destinés aux personnes âgées isolées physiquement ou socialement au Canada, dans les années à venir.

## Expériences et défis en matière de soins de longue durée

### Veronique Boscart, Michelle Heyer, et Linda Sheiban Taucar

Les personnes âgées, notamment celles qui vivent dans les établissements de soins de longue durée (SLD), sont sévèrement touchées par la propagation de la COVID-19. Les cas de COVID-19 sont plus graves chez les personnes souffrant de morbidités multiples ou dont le système immunitaire est affaibli. Les infections virales peuvent donc se développer beaucoup plus rapidement dans ces populations (Rothan & Byrareddy, 2020). Les résidents et leurs soignants se retrouvent infectés, entraînant un taux de mortalité accru chez les plus vulnérables. Les décès résultant de la COVID-19 dans les établissements de SLD représenteraient environ 63 % de l’ensemble des décès dus à la COVID-19 au Canada (Hsu & Lane, 2020). En Ontario uniquement, plus de 150 établissements de SLD ont connu des éclosions de COVID-19 (Gouvernement de l’Ontario, 2020). Ces éclosions affectent non seulement la santé physique générale des individus, mais aussi leur sphère sociale. Les centres de SLD limitent désormais les visites, accroissant l’isolement social des résidents. En raison de troubles cognitifs, certains résidents ne peuvent comprendre pourquoi leurs proches ne leur rendent pas visite, ce qui génère de l’anxiété et un sentiment de solitude. Le Gouvernement du Canada (2020a) a entrepris de repérer le personnel soignant travaillant dans plus d’un établissement et de restreindre cette pratique, ce qui devrait contribuer à ralentir la propagation de la COVID-19. En outre, dans certains centres de soins de longue durée, on constate une pénurie de personnel. Dans les centres où le personnel est insuffisant, une augmentation des niveaux d’épuisement professionnel pourrait survenir. Des ressources appropriées seront nécessaires afin de garantir que les personnes qui sont affectées par la COVID-19 — sur le plan physique, psychologique, comportemental et social — soient en mesure de rechercher et de recevoir un soutien approprié.

## Le « grand égalisateur » ? La précarité aux temps de la pandémie

### Catherine Tong

Malgré la notoriété de la COVID-19 dans la culture populaire, cette dernière ne constitue pas le « grand égalisateur » (Owoseje, 2020). En effet, toutes les personnes ne bénéficient pas, au cours de cette pandémie, des mêmes avantages et des mêmes ressources. Bien que les efforts soient actuellement concentrés, à juste titre, sur les soins de longue durée, la pandémie met aussi en évidence la précarité des personnes âgées qui reçoivent des soins à domicile et d’autres formes de soutien communautaire. Les chercheurs ont depuis longtemps sonné l’alarme sur les conditions des travailleurs des services d’aide à domicile qui fournissent des soins dans les milieux privés non réglementés. Ces travailleurs visitent ainsi jusqu’à une douzaine de résidences par jour, et travaillent en grande partie seuls (Craven et al., 2012 ; Doran et al., 2009 ; Macdonald & McLean, 2018). Étant donné la nature précaire de leur travail (Zagrodney & Saks, 2017), de nombreux travailleurs du soutien à domicile œuvrent à la fois en institutions de soins de longue durée et dans les soins à domicile, bien que certaines provinces aient suspendu cette pratique depuis l’arrivée de la COVID-19 (DeClerq, 2020 ; Eagland, 2020).

Par ailleurs, partout au Canada, les proches aidants sont touchés par la COVID-19. Pour ceux qui tiennent à l’engagement des patients et de leur famille (Change Foundation, 2016), il est inquiétant de constater que les proches aidants, auparavant considérés comme des « partenaires de soins », ne sont plus autorisés à entrer dans de nombreux établissements, bien que des membres de leur famille y soient placés en soins intensifs. Ils doivent naviguer dans un système de soins de santé qui a rapidement changé et qui, dans le pire des cas, recevra leur dernier souffle. Pour de nombreux proches aidants, ces expériences se produisent alors qu’ils doivent simultanément composer avec une multiplicité de rôles et de responsabilités (p. ex. soignant, employé, parent), tout cela à distance. Ainsi, les aidants familiaux sont confrontés à un niveau de précarité encore plus élevé, à des demandes nouvelles et uniques dues à la COVID-19, qui auront des répercussions à long terme pour les proches aidants et le système de soins de santé en général.

Un autre type de précarité exacerbé par la pandémie est associé à l’impact profond et potentiellement disproportionné de celle-ci sur les personnes appartenant à des minorités visibles ou ethnoculturelles. Près d’un Canadien âgé sur sept fait partie d’une minorité visible (Statistique Canada, 2016), et présenterait une vulnérabilité accrue à la COVID-19 en raison de l’âge, de l’appartenance ethnique et d’autres dimensions (Hankivsky, 2011). Bien que certaines villes, comme la ville de Toronto, aient choisi de collecter des données sur l’ethnicité dans le cadre de leurs suivis concernant la COVID-19 (Mojtehedzadeh, 2020), il n’est pas prévu de le faire à l’échelle fédérale (Nasser, 2020). Pour mieux comprendre l’impact de la COVID-19 sur les minorités visibles ou ethnoculturelles plus âgées, les chercheurs devront faire appel à une vaste gamme d’approches méthodologiques et à des techniques adaptées au contexte culturel (Liamputtong, 2010).

Collectivement, la communauté scientifique devra s’engager significativement auprès des groupes qui font face à cette pandémie dans des conditions précaires. Bien qu’il soit crucial d’identifier les individus et les groupes qui sont confrontés à des inégalités systémiques et persistantes, il est tout aussi important de reconnaître et de mettre en évidence la résilience et les forces de ces communautés. Les approches basées sur les forces (Moyle, Parker, & Bramble, 2014), la recherche collaborative (Marlett et Emes, 2010), et la co-conception authentique (Donetto, Tsianakas, & Robert, 2014) permettront à notre communauté de chercheurs en gérontologie de mieux comprendre et d’élaborer une réponse adaptée à cette pandémie, en demeurant particulièrement attentifs à ceux qui subissent une précarisation accrue en raison de la COVID-19.

## Les aspects sociaux et culturels du vieillissement et de la santé ne doivent pas être oubliés

### Christine Kelly

La COVID-19 n’est pas une maladie équitable. Bien entendu, les personnes âgées, handicapées et celles souffrant de problèmes de santé sous-jacents sont plus à risque de souffrir de formes plus graves de COVID-19. Toutefois, il semble également que la COVID-19 affecte de manière disproportionnée les personnes non blanches (Ormiston, 2020). Cette réalité en évolution met en évidence la manière dont les structures systémiques d’exclusion et de marginalisation se manifestent par une augmentation des risques et de l’oppression pendant la pandémie mondiale. Par conséquent, nous nous devons d’examiner spécifiquement comment l’âgisme, la discrimination fondée sur la capacité physique, le racisme et d’autres formes d’oppression systémique façonnent les discours publics liés à la COVID-19. Il est essentiel de prendre en compte ces questions sociales plus larges dans le cadre de l’étude et de la réponse à la COVID-19. Le contexte social ne peut être écarté des réponses physiologiques et politiques à cette situation.

À l’heure actuelle, les milieux de recherche et les médias accordent une importance majeure aux essais cliniques et à la recherche fondée sur l’intervention contre la COVID-19. Toutefois, même les domaines les plus centrés sur l’approche médicale devraient prendre en considération les facteurs sociaux, en incluant des spécialistes des sciences sociales et humaines, et en ayant recours à des outils théoriques appropriés et critiques, tels que l’intersectionnalité (Rice, Harrison, & Friedman, 2019), l’équité en matière de santé (Marmot & Allen, 2014) et la précarité (Grenier, Lloyd, & Phillipson, 2017). Ce sont là quelques exemples des nombreux points de départ potentiels pour comprendre les structures sociales plus vastes qui influencent les contextes marqués par la COVID-19 pour les diverses populations de personnes âgées. Outre l’ajout d’une optique sociale à la recherche médicale, il est nécessaire de mener des recherches employant des méthodes qualitatives qui permettent de recueillir les différentes perceptions et comptes rendus d’expériences vécues dans ces contextes. Ces recherches exigeront une adaptation créative pour respecter les directives en matière de distanciation physique.

Finalement, la gérontologie culturelle, en tant qu’application élargie des sciences humaines dans le champ du vieillissement, a apporté des contributions significatives à notre compréhension de l’âge et du vieillissement (Twigg & Martin, 2015). Plus important encore, ce type de travaux peut générer de nouvelles orientations théoriques et contribuer à une analyse en profondeur des représentations artistiques de notre époque, telles qu’elles se manifesteront dans la musique, la littérature, le cinéma et les autres médias. Beaucoup d’entre nous constateront un essor des arts créatifs — poésie, danse, musique, arts visuels — tous inspirés par ce moment inhabituel de l’histoire. Nous nous tournons vers les arts pour y trouver une source de bien-être, ce qui indique que les arts créatifs, et notre analyse de ceux-ci, ne sont pas des accessoires, mais plutôt des éléments essentiels à l’appréciation de la réalité actuelle. Pendant cette période extraordinaire, les chercheurs, les praticiens et les autres intervenants sont invités à ne pas oublier les aspects sociaux et culturels de la santé et du vieillissement.

## Saisir l’importance de la perspective des personnes âgées sur la vie pendant la pandémie

### Deborah K. van den Hoonaard

Bien que la COVID-19 apparaisse essentiellement comme une problématique liée à la santé, il est nécessaire d’effectuer des recherches approfondies pour nous aider à mieux comprendre la signification sociale et les expériences vécues par les personnes âgées lors de cette pandémie. Nous devrons nous poser des questions sur les représentations de personnes âgées dans les médias, et dans les discours des politiciens et des experts en santé. L’utilisation des termes « personnes âgées » donne l’impression que toutes les personnes âgées se ressemblent, même si elles forment un groupe très hétérogène (Applewhite, 2016 ; Gullette, 2004). La réémergence de l’expression « tsunami gris » (*grey tsunami*) est aussi troublante. Pour considérer la population âgée comme pleinement humaine, il est nécessaire de confronter la « hiérarchie de la crédibilité » (Becker, 1967; van den Hoonaard, 2018), qui accorde une priorité aux opinions des « experts » plutôt qu’à celles des personnes âgées. Il sera important pour nous de comprendre comment la vie quotidienne des personnes âgées a changé pendant cette période. Les sociologues et les spécialistes du discours pourront élaborer une « histoire collective » qui permettra de « faire entendre la voix de ceux dont les récits ont été exclus du domaine public et du discours citoyen, rendant ainsi possible l’émergence d’une identité collective et de solutions collectives » (Richardson, 1990, p. 28). Il sera important, dans les prochaines années, de réaliser des recherches inductives et qualitatives qui permettront de connaître le point de vue des personnes âgées concernant l’impact de la COVID-19 sur leur vie. En tant que gérontologues, nous avons tendance à considérer les personnes âgées comme des personnes vulnérables. Cependant, cette perspective est remise en question lorsque nous tenons compte de leurs points de vue, de leur créativité et de leur résilience, et lorsque nous adoptons une optique plus globale, en fonction de laquelle la santé n’est pas le seul élément à prendre en compte, même en temps de pandémie.

## Points de vue critiques sur le vieillissement et la COVID-19 : une expertise gérontologique accrue est nécessaire

### Brad A. Meisner, Ariane S. Massie, et Laura Kadowaki

Les Canadiens sont inondés par les campagnes de santé publique locales et mondiales relatives à la COVID-19. Il convient d’examiner comment ces campagnes ont contribué ou affecté la compréhension du vieillissement et des personnes âgées. Il est désormais bien connu que les risques d’effets néfastes sur la santé sont sensiblement plus élevés chez les personnes âgées que chez les enfants, les jeunes et les jeunes adultes. Tant le Gouvernement du Canada (2020b) que l’Organisation mondiale de la Santé (2020) identifient les personnes âgées comme étant le groupe le plus exposé aux graves conséquences de la maladie, aux hospitalisations, aux soins intensifs et au décès en raison de la COVID-19. Cependant, l’utilisation et la représentation de l’âge chronologique dans ces messages médiatiques peuvent être critiquées de plusieurs points de vue. Tout d’abord, l’âge est catégorisé à partir de valeurs limites, le groupe des « adultes âgés » étant défini comme celui des personnes âgées de 60 ou 65 ans et plus (respectivement selon l’Organisation mondiale de la Santé, 2020 ; Gouvernement du Canada, 2020b).

Du point de vue de la durée de vie, l’utilisation de seuils liés à l’âge chronologiques est arbitraire et doit être évitée, car elle représente faussement les personnes âgées comme un groupe homogène (Ayalon et al., 2020). Ces seuils ne tiennent pas compte des différences et des diversités biologiques, psychologiques, sociales et écologiques qui existent entre les personnes âgées, particulièrement lorsque la classification selon l’âge englobe plusieurs décennies. Deuxièmement, les messages diffusés sur les risques liés à la COVID-19 sont imputés à l’âge chronologique, alors des facteurs autres que l’âge semblent plus fortement associés à ce risque (Montero-Odasso et al., 2020). Par exemple, bien que les taux d’hospitalisation soient plus élevés chez les personnes âgées, une grande proportion des patients hospitalisés présente des problèmes de santé sous-jacents, quel que soit leur âge (Garg et al., 2020). Troisièmement, l’utilisation de seuils arbitraires pour l’âge et l’attribution (erronée) généralisée du risque de COVID-19 au vieillissement ont, involontairement, renforcé et intensifié les stéréotypes négatifs sur l’âge, les préjugés et la discrimination, qui devront désormais être pris en considération (Ayalon et al., 2020 ; Brooke & Jackson, 2020 ; Meisner, 2020). Même avant la pandémie, les interventions visant à réduire l’âgisme étaient importantes et il convient donc de les renforcer davantage (Burnes et al., 2019).

Pour faire face à l’âgisme et discerner les faits réels des fictions en matière de COVID-19 et de vieillissement, il est nécessaire d’augmenter le nombre et la participation des professionnels ayant une formation et une expertise en gérontologie. Actuellement, au Canada, la formation en gérontologie au premier cycle est insuffisante, et peu de programmes d’études supérieures sont offerts dans ce domaine (Wister, Kadowaki, & Mitchell, 2016). Les établissements d’enseignement supérieur pourraient devoir réévaluer leur approche en matière de recrutement d’étudiants pour ces programmes. La perception des étudiants sur le travail avec les personnes âgées est généralement négative, ce qui les dissuade de poursuivre des études supérieures dans le domaine du vieillissement ou d’y envisager une carrière (Algoso et al., 2016). Étant donné l’intensification du discours âgiste associé à la COVID-19 (Meisner, 2020), il est peu probable que cette tendance soit modifiée, et encore moins améliorée. Une fois inscrits dans les programmes de gérontologie, les étudiants ont besoin d’occasions d’apprentissage efficaces. La formation et les interactions de qualité avec des personnes âgées au moyen de l’apprentissage expérientiel permettent de réduire les préjugés négatifs liés à l’âge (Obhi et Woodhead, 2016) et augmentent l’intérêt pour le travail avec des personnes âgées (Allen et al., 2014). Ces expériences d’apprentissage doivent être adaptées (p. ex. interactions virtuelles) pour répondre aux urgences de santé publique actuelles et potentielles. La présente crise incite à investir davantage dans l’enseignement de la gérontologie. Elle s’avère aussi une occasion pour reconfigurer les programmes d’enseignement afin d’y inclure des problématiques importantes, visant l’amélioration des conditions de la population vieillissante et de la société, dans une perspective s’étendant au-delà de la pandémie.

## Recherche sur la COVID-19 et le vieillissement au Québec

### Mélanie Levasseur et Pierrette Gaudreau

Le Québec a un profil particulier au Canada en ce qui concerne la COVID-19. En date du 7 mai, 54 % des cas de COVID-19 et 60 % des décès au Canada étaient survenus au Québec (Gouvernement du Canada, 2020c ; Institut national de santé publique du Québec, 2020). Considérée comme l’épicentre de la COVID-19 au Canada, la ville de Montréal à elle seule a compté 17 918 cas confirmés et 1 666 décès (Gouvernement du Québec, 2020). Comme dans d’autres provinces et pays, les décès sont surreprésentés chez les personnes âgées de 80 ans et plus. En réponse à ces tendances et données alarmantes, le Réseau québécois de recherche sur le vieillissement a lancé un appel à propositions sur la COVID-19 avec le soutien financier du Fonds de recherche du Québec-Santé. Trois thèmes prioritaires de recherche concernant la COVID-19 ont été identifiés dans le cadre de ce concours provincial, qui porte sur les perspectives biologique, psychologique, sociale et sociétale.

Le premier thème inclut des projets qui visent à améliorer la qualité de vie et les expériences des personnes âgées dans différents environnements physiques et sociaux affectés par la COVID-19 (p. ex. SLD, milieux communautaires), en considérant aussi les personnes âgées souffrant d’un handicap physique ou de détresse psychologique et celles qui sont confrontées à l’isolement social ou à un manque de soins. Le deuxième thème comprend des projets visant à développer, mettre en œuvre et évaluer de nouveaux outils et stratégies pour préserver l’état de santé, la mobilité fonctionnelle et la qualité de vie des personnes âgées touchées par la COVID-19. Le troisième thème vise les projets cherchant à organiser et à évaluer l’accessibilité des systèmes de soins de santé et les stratégies utilisées par les organisations communautaires ou prestataires des soins, pour adapter la prestation de services de soutien aux personnes âgées et aux aidants en période de distanciation physique et d’isolement social. Les recherches financées dans le cadre de ces thèmes représentent un élément de la réponse du Québec à la COVID-19 et à son impact sur le vieillissement et les personnes âgées. Les résultats de ces projets permettront de traiter des enjeux et d’identifier les avenues prometteuses pour la promotion de la santé et de la qualité de vie des personnes âgées vivant dans divers environnements et présentant des conditions de santé variées. Les résultats amélioreront notre compréhension collective des impacts de la COVID-19 et notre préparation pour de futures situations d’urgence publique.

## La COVID-19 et l’Étude longitudinale canadienne sur le vieillissement

### Verena Menec

L’Étude longitudinale canadienne sur le vieillissement (ELCV) est particulièrement bien positionnée pour fournir une plateforme de données pour la recherche interdisciplinaire sur les complexités et les conséquences de la COVID-19 sur les personnes âgées et le vieillissement au Canada. L’ELCV est une initiative stratégique nationale des Instituts de recherche en santé du Canada qui vise à comprendre divers sujets et questions liés au vieillissement (Kirkland et al., 2015 ; Raina et al., 2009 ; Raina et al., 2019). Lancée en 2010, l’étude incluait au départ 51 339 Canadiens âgés de 45 à 85 ans. Les participants ont effectué leur premier suivi entre 2015 et 2018, et le deuxième suivi est en cours. Les chercheurs ont accès aux données des entretiens, des évaluations physiques et des biomarqueurs par un processus de demande d’accès aux données (voir https://www.clsa-elcv.ca). En réponse à la pandémie actuelle, l’équipe de l’ELCV a lancé une sous-étude portant sur la COVID-19 en avril 2020, en menant des entretiens en ligne et par téléphone. Ces entretiens permettent de recueillir des informations sur les symptômes, les facteurs de risque, l’utilisation des soins de santé et de nombreux autres éléments importants associés à la COVID-19, à l’éloignement physique et à son influence sur la santé mentale. Le questionnaire de base sur la COVID-19 sera suivi par des questionnaires hebdomadaires, bihebdomadaires et mensuels pendant une période de six mois. Les données spécifiques sur la COVID-19 seront disponibles et pourront être utilisées en combinaison avec les informations déjà disponibles dans l’ELCV. L’ELCV constituera ainsi une source inédite de données permettant d’identifier et de se pencher sur certains enjeux importants et sur les opportunités mises en évidence dans la présente déclaration et d’autres projets en cours.

## L’expérience naturelle mondiale

### Paul Stolee

La COVID-19 est une pandémie planétaire et, bien que pratiquement tous les pays du monde aient déclaré des cas d’infection avec ce virus, la réaction des différentes juridictions a considérablement varié (Hale et al., 2020). Les réponses à la pandémie sont déterminées en partie par les données et les modèles mathématiques, mais aussi par les contextes politiques (Wenham, 2020). La plupart des pays ont adopté des mesures qui imposent l’éloignement physique. Cependant, la Suède a résisté de façon controversée à une approche de « confinement » (Ward, 2020). La réponse fédérale des États-Unis et certaines variations des réponses régionales ont aussi été très contestées (Lasry et al., 2020 ; Villarreal, 2020). Inversement, certains dirigeants, tant au niveau international qu’au Canada, ont été félicités pour avoir réagi de manière plus efficace. Au niveau international, la Nouvelle-Zélande fait office d’exemple (Friedman, 2020). Au Canada, les mesures de santé publique prises à temps par la Colombie-Britannique ont permis de mieux « aplatir la courbe » (Mason, 2020). Les variations nationales et internationales dans les réponses de santé publique et sociétale à la COVID-19 sont décrites comme une « expérience naturelle globale » (Thomson, 2020). Les différents niveaux des juridictions publiques sont considérés comme des « laboratoires » (Karch, 2007), puisqu’ils ont le pouvoir de tester différents types de programmes et de politiques d’intervention.

Le système fédéral canadien fait en sorte que les réponses politiques et de santé publique à la COVID-19 peuvent varier entre les niveaux national, provincial et territorial, ainsi qu’au niveau municipal (Meekison, 1977 ; Simeon, 2006). Toutes les juridictions ont signalé la présence de cas de COVID-19 (à l’exception du Nunavut, à ce jour) et ont mis en place des mesures strictes en matière de distanciation physique. Cependant, encore là, leur mise en œuvre et celle des autres mesures de santé publique sont variables et temporaires. Par exemple, l’accent a été mis sur la préparation du système de soins intensifs, possiblement au détriment des soins à domicile et communautaires. Au début du mois de mars 2020, des experts en politique publique ont réclamé que des lits d’hôpitaux soient libérés par le transfert de patients vers les soins à domicile ou les soins de longue durée (Forest & Sutherland, 2020). À la mi-mars 2020, le gouvernement de l’Ontario a annoncé qu’il prendrait « toutes les précautions nécessaires pour garantir la sécurité des personnes vivant dans les établissements de soins de longue durée de l’Ontario » (Crawley, 2020), alors qu’aucun décès n’avait encore été signalé dans les centres de SLD ontariens. Cependant, à la mi-avril 2020, près de la moitié des décès dus à la COVID-19 enregistrés au Canada étaient liés aux centres de SLD (Aiello, 2020). Le 22 avril, le gouvernement ontarien a demandé l’aide des forces armées canadiennes dans cinq centres de SLD de l’Ontario (Crawley, 2020). Par ailleurs, à la mi-avril 2020, le gouvernement fédéral a publié des directives de santé publique pour les centres de SLD (Agence de la santé publique du Canada, 2020), et le gouvernement ontarien a produit un plan d’action destiné aux centres de SLD pour faire face à la COVID-19 Ministre de la Santé et des Soins de Longue Durée de l’Ontario, 2020).

Aucune personne n’aurait envisagé ou demandé des occasions d’apprentissage comme celle associée à la COVID-19. Cependant, la pandémie a mis en lumière les lacunes de notre société en matière de soutien aux personnes âgées. Le coût de cette négligence peut se mesurer par les taux alarmants d’infection et de mortalité chez les personnes âgées, comme cela a été tragiquement observé dans une résidence privée pour aînés à Dorval, au Québec (Feith, 2020). Il n’était pas inévitable que cela se produise, puisque les premières réponses mises en place dans les centres de SLD ont permis d’éviter des éclosions dans certaines régions (Howlett, 2020). Il est donc impératif que nous prenions conscience de « l’expérience naturelle » associée aux différentes réponses politiques et de santé publique qui ont lieu dans les multiples « laboratoires » des diverses juridictions. Il est particulièrement important que nous utilisions cette expérience afin d’améliorer les soins, les conditions et les résultats pour les personnes âgées et les populations vulnérables vivant en centres de SLD (p. ex. Armstrong et al., 2020). Cette initiative doit débuter dès maintenant, pour être en mesure de surveiller les réponses politiques et de santé publique à la COVID-19 qui évoluent de jour en jour, et pour mieux comprendre les expériences des personnes âgées touchées, ainsi que celle de leurs familles, durant et après la pandémie. Il est également crucial que ce travail soit abordé de façon holistique et qu’il prenne en compte les conséquences sanitaires et sociales de la COVID-19, ainsi que les faiblesses préexistantes du système, en vue d’assurer le soutien du bien-être physique, mental et social des Canadiens âgés.

## Conclusion

Dans les sections précédentes de cette déclaration commune de l’ACG/CAG et de la *RCV/CJA*, nous avons examiné quelques-unes des principales questions et possibilités associées à la perspective gérontologique qui peuvent être considérées en réponse à la COVID-19. Bien que le cadre de cette déclaration puisse limiter le nombre de sujets présentés, les exemples mentionnés illustrent l’importance des approches interdisciplinaires en cette période de changements personnels et sociétaux majeurs. Comme nous l’avons montré dans les différentes sections, la recherche en gérontologie et la pratique appliquée à la COVID-19 doivent être considérées de façon globale. Les perspectives multiples et l’intersection de différentes disciplines génèrent une valeur ajoutée comparativement à la recherche s’appuyant sur une seule discipline.

Puisqu’une seule discipline et une seule personne ne pourront expliquer entièrement ce phénomène, la collaboration est donc indispensable. Nous préconisons le recours à des équipes multidisciplinaires qui rassemblent et rapprochent différents domaines d’expertise, ainsi que l’utilisation de méthodes de collecte d’informations, de données, d’évaluation et de communication qui soient multiples. Grâce à la combinaison de ces stratégies, nous pourrons travailler conjointement sur des sujets interdisciplinaires afin d’évaluer d’une façon critique et globale comment les personnes âgées et la population vieillissante sont touchées par la COVID-19. Nous encourageons chacun d’entre vous — chercheurs, professionnels, représentants d’organisations, étudiants, soignants et personnes âgées — à considérer vos disciplines, professions et communautés respectives et à réfléchir aux moyens qui vous permettront de former des équipes multidisciplinaires et d’y contribuer. Lorsque vous serez prêts à diffuser vos travaux, nous vous invitons à répondre à notre prochain appel à communications sur la COVID-19 dans la *Revue canadienne du vieillissement/Canadian Journal on Aging.* Dans l’attente, nous espérons que cette déclaration vous fournira des pistes et des orientations utiles dans la poursuite de nos efforts communs liés à la COVID-19, dans une perspective de points de vue multiples en gérontologie.
